# Vocal effectiveness of speech-language pathology students: Before and after voice use during service delivery

**DOI:** 10.4102/sajcd.v62i1.95

**Published:** 2015-03-26

**Authors:** Stephanie Couch, Dominique Zieba, Jeannie van der Linde, Anita van der Merwe

**Affiliations:** 1 Department of Speech-Language Pathology, University of Pretoria, South Africa

## Abstract

**Background:**

As a professional voice user, it is imperative that a speech-language pathologist's (SLP) vocal effectiveness remain consistent throughout the day. Many factors may contribute to reduced vocal effectiveness, including prolonged voice use, vocally abusive behaviours, poor vocal hygiene and environmental factors.

**Objectives:**

To determine the effect of service delivery on the perceptual and acoustic features of voice.

**Method:**

A quasi-experimental., pre-test–post-test research design was used. Participants included third- and final-year speech-language pathology students at the University of Pretoria (South Africa). Voice parameters were evaluated in a pre-test measurement, after which the participants provided two consecutive hours of therapy. A post-test measurement was then completed. Data analysis consisted of an instrumental analysis in which the multidimensional voice programme (MDVP) and the voice range profile (VRP) were used to measure vocal parameters and then calculate the dysphonia severity index (DSI). The GRBASI scale was used to conduct a perceptual analysis of voice quality. Data were processed using descriptive statistics to determine change in each measured parameter after service delivery.

**Results:**

A change of clinical significance was observed in the acoustic and perceptual parameters of voice.

**Conclusion:**

Guidelines for SLPs in order to maintain optimal vocal effectiveness were suggested.

## Introduction

The vocal health and voice management of professional voice users has gained increasing interest in recent years: professional voice users are considered to be at risk of developing voice problems and complaints (Van Lierde *et al*., 2008). Since the professional voice users rely on their voices as the means to carry out their occupational duties, their voices can be described as a ‘primary tool of trade’ (Warhurst, Madill, McCabe, Heard & Yiu, 2010; Titze, Lemke & Montequin, 1997). Considering the importance of voice to professional voice users, it is imperative that their voices remain clear and stable throughout the day. Less than optimal vocal effectiveness (pitch, loudness, quality and flexibility) may negatively influence the professional voice user's ability to carry out their daily occupational demands (Hazlett, Duffy & Moorhead, 2009; Roy, Merril, Thibeault, Gray & Smith, 2004). This highlights the need for the prevention, identification and treatment of voice problems before they become significant enough to influence occupational duties negatively.

Professional voice users are amongst the most likely individuals to develop vocal pathologies or voice disorders as a result of improper or excessive voice use (Broaddus-Lawrence *et al*., 2000). They often demonstrate excessive amounts of voice use in sometimes non-optimal environments as part of their occupational requirements or demands (Colton, 2011). Occasional vocal abuse or misuse may not have a significant impact on vocal parameters, however, the chronic use of vocally abusive behaviours may result in vocal problems such as hoarseness, reduced pitch range, vocal fatigue and the sensation of pressure, tightness or pain in the throat (Ng, Bailey & Lippert, 2005). Given the vocal demands placed on the professional voice user's voice, it is almost expected that vocal problems may manifest. Pathologies on the vocal folds may occur as a result of vocal overuse, chronic voice abuse and misuse (Johns, 2003). Organic manifestations such as nodules, cysts and granuloma may develop after initial wounds have healed which resulted in the remodelling of the superficial layer of the lamina propria. Lesions on the vocal folds, such as nodules, result in mucosal wave disruption during phonation (Johns, 2003). This in turn may result in perturbation of the acoustic wave. In an attempt to overcome the resulting change in voice parameters, the individual may attempt to compensate by making use of excessive effort, abuse and misuse of the voice. Subsequently, a vicious cycle of vocal misuse and abuse may ensue in order to compensate for the loss of vocal effectiveness. Some of the most common consequences of voice problems may include a change in job performance and an absence from work (Yiu, 2002; Roy *et al*., 2004).

The SLP is a professional voice user with a very specialised set of voice use requirements. The occupational vocal demands that SLPs experience daily are profession-specific (Warhurst *et al*., 2010). These demands may be a significant causal factor in the development of vocal difficulties. Increasing vocal intensity or loudness whilst maintaining a clear voice quality, as well as maintaining appropriate vocal power, stamina and control are, amongst other conditions, categorised as vocal demands (Timmermans, De Bodt, Wuyts & Van de Heyning, 2004; Warhurst *et al*., 2010). SLPs may use an increasing number of vocally demanding behaviours to keep the client's attention in therapy; for instance, using auditory highlighting. Voice therapy particularly requires not only the proper use of voice to be demonstrated to clients who are receiving therapy for their voice disorder but also incorrect vocal behaviours. SLPs use their voices in a variety of ways for a range of additional purposes. These purposes may include public speaking, counselling, conferencing, therapy and class presentations in the school context (Gottliebson, Lee, Weinrich & Sanders, 2006). Therefore, as the profession that treats voice disorders and that has a role in advocating vocal health, it is essential that the speech-language pathologist prioritise vocal health and adequate vocal effectiveness.

According to Gottliebson *et al*. (2006), a lower prevalence of voice disorders occurs in SLP students than in other students; however, a small number of SLP students are still identified as having a voice disorder. For these students, the resolution of their difficulties will be essential to a ‘successful professional voice career’ (Gottliebson *et al*., 2006, p. 704). The purpose of this study is to determine whether two hours of voice use during service delivery (e.g. providing therapy to an individual client) has an effect on the voice characteristics of a group of SLP students. It is possible that both the perceptual and the acoustic features of voice will demonstrate a diminished effectiveness as a result of voice use during service delivery.

## Method

### Research design

A quasi-experimental, pre-test–post-test design was applied.

#### Participants

Purposive sampling was used and 12 participants (see Table 1) were selected according to the pre-set selection criteria: participants were required to be female and between the ages of 18 and 30 years. Participants were excluded if they were smokers and had a history of voice disorders or gastro-oesophageal reflux disease. The population included third- and final-year SLP students enrolled at a university in South Africa.

**TABLE 1:** Description of participants’ characteristics.

Participant	Age	Allergies	Sinusitis	Post-nasal drip	Ear problems	Chronic colds	Asthma	Mouth breathing	Tonsillitis	Tonsillectomy	Thyroid surgery	Adenoidectomy	Endocrine illnesses/hormonal treatment	Laryngeal injury/anesthetic	Other medical problem	Medication	Birth control
2	23		x	x						x							x
3	21																
4	23	x					x										
5	22		x	x						x							
7	22		x	x		x		x									x
9	20	x	x														
10	21		x					x									x
11	25																
12	23																x
13	21																
14	22	x															
15	25		x													x	

Information letters were provided to each participant and informed consent was given by all the participants in the study. No risks were involved in the study.

### Protocol used for data collection

A short case history was obtained and three assessment instruments were used. The data collected in the case history included a search for factors that may have a negative influence on vocal effectiveness. The assessment instruments included the GRBASI 4-point scale (see Table 2) to rate perceptual data (Yamauchi, Imaizumi, Maruyama & Haji, 2010), the Dysphonia Severity Index (DSI) (Wuyts *et al*., 2000), a quantitative correlate of vocal quality, maximum phonation time and the s/z ratio (Shipley & McAfee, 2009). The Voice Range Profile (VRP) and the Multidimentional Voice Program (MDVP) software packages (KayPENTAX, 2008) were used to measure the vocal parameters necessary to calculating the DSI, including shimmer, jitter, highest frequency, lowest intensity, fundamental frequency and noise to harmonics ratio (see Table 3).

**TABLE 2:** GRBASI 4-point rating scale.

Component		Description
G	Grade	Degree of hoarseness of the voice
R	Roughness	Impression of irregularity of the vibration of the vocal folds
B	Breathiness	Degree to which air escaping from between the vocal folds can be heard by the examiner
A	Asthenia	Degree of weakness heard in the voice
S	Strain	Extent to which strain or hyperfunctional use of phonation is heard
I	Instability	Changes in voice quality over time

Rating scale: 0, normal; 1, slight; 2, moderate; 3, severe.

*Source*: Yamauchi, E.J., Imaizumi, S., Maruyama, H. & Haji, T. (2010). Perceptual evaluation of pathological voice quality: A comparative analysis between the RASATI and GRBASI scales. *Logopedics Phoniatrics Vocology*, *35*, 121–128.

**TABLE 3:** Vocal parameters measured.

Vocal Parameter	Description
Highest frequency	Highest frequency in Hz is obtained from phonation of /a/ at a comfortable or habitual pitch and loudness (Timmermans et al., 2004). For the purposes of this study frequency and pitch are defined as “the number of vibratory cycles per second” and the sensation of that as perceived by the listener respectively (Raphael, Borden & Harris, 2007: p. 44).
Lowest intensity	Lowest intensity in dB is obtained from phonation of /a/ at a. comfortable or habitual pitch and loudness (Timmermans et al., 2004). For the purposes of this study, intensity is defined as the physical property of sound pressure of the acoustic signal, whereas loudness is the sensation of the perceived sound pressure by the listener (Raphael, Borden & Harris, 2007).
Maximum phonation time	Phonation of /a/ for as long as possible at a comfortable pitch and loudness, after maximum inspiration, is recorded in seconds and milliseconds. Each participant is allowed three trials, after which the best result is recorded (Dejonckere *et al*., 2001; Timmermans *et al*., 2004).
Jitter	The average difference in frequency (pitch) from cycle to cycle of a sound wave over time or the instability of pitch. Jitter is stated as a percentage (Dejonckere *et al*., 2001; Schaeffer, 2012).
Shimmer	The average difference or instability in amplitude (loudness) of a sound wave over time (Dejonckere *et al*., 2001; Schaeffer, 2012).
Noise/harmonics ratio	The concentration of energy around the harmonics (overtones) of a sound or the ratio of the noise to the vocal signal; this reflects irregular vocal fold vibration (Warhurst *et al*., 2010; Schaeffer, 2012).
Fundamental frequency	The habitual frequency (in Hz) at which the vocal cords vibrate (Haynes & Pindzola, 2012).

### Procedures

Each participant completed a case history. Participants were instructed not to drink caffeinated drinks or alcoholic beverages 12 hours prior to the first assessment to ensure the use of these did not influence their voice quality. Audio recordings of the voices of each participant were made. Participants were instructed to hold the microphone at a fixed distance of 30 cm from the mouth to obtain the most consistent results across the different participants and to obtain optimal quality recordings (Van Lierde *et al*., 2008; Timmermans *et al*., 2004).

Voice recordings for acoustic analysis were made in a sound-proof room. The recording facility of the computerised speech lab (or CSL) (KayPENTAX, 2008) was used to make the recordings. Participants were asked to sustain phonation of the /a/, /i/ and /u/ sounds three times each at a comfortable pitch and loudness. The /a/ vowel was used to record the highest and lowest frequency and the highest and lowest intensity using the VRP. The participant was instructed to produce highest and lowest pitch. A stop-watch was used to record the maximum phonation time of the participant using the /a/ sound. The s/z ratio was recorded by using the stop-watch.

Participants completed two hours of speech therapy individually in similar noise-controlled room conditions. The type of therapy provided varied between participants and included adults and children. Noise levels in surrounding rooms and corridors were kept to a minimum to prevent this factor from negatively influencing the results. This helped to control the impact of treating adults and children. High noise levels in the environment may also negatively influence vocal parameters (Lehto *et al*., 2006).

The post-test was done once the participants had completed the experimental condition (providing two consecutive hours of therapy) and before any amount of voice rest was possible. Voice rest may result in an improved quality of voice (Van der Merwe, 2004). The same procedures were performed as during the pre-test.

### Quantitative data analysis

The perceptual data of the participants’ voice quality was collected following the GRBASI scale (Table 2). A listener panel consisting of three judges took part in the analysis. To ensure reliable data all of them were experienced listeners (Howard & Lohmander, 2011). The sentence recordings were played back in random order to remove bias. Each member of the panel of listeners gave a rating for each component of the GRBASI scale. Consensus was then reached by majority vote for each component.

The DSI was calculated from the measurements collected by the MDVP, VRP and the maximum phonation time. The DSI is interpreted as follows: scores may range from –5 to +5. A score of +5 indicates a perceptually normal voice whereas a score of –5 indicates a voice that is severely dysphonic. The degree of dysphonia as measured by the DSI represents the optimal combination of independent variables to correspond with the G component of the GRBASI scale (Timmermans *et al*., 2004; Wuyts *et al*., 2000). In Table 3 the vocal parameters measured were presented. Changes in the acoustic data were related to the presence or absence of participant characteristics (collected in the case history) that may have been contributors to reduced vocal effectiveness.

### Data processing

The perceptual data were processed by comparing the scores obtained for each participant in the pre- and post-tests. For each component within the GRBASI scale, values may range from 0–3 (where 0 = normal, 1 = slight, 2 = moderate, 3 = severe).

The acoustic data were analysed by means of descriptive statistics using STATA 12. Descriptive statistics consisted of calculations of medians and range for each acoustic value (shimmer, jitter, highest frequency, lowest intensity, fundamental frequency and noise to harmonics ratio) collected before and after the experimental condition. The sign test was used to determine whether the median for acoustic values differed from the pre-test to the post-test results (Dixon & Mood, 1946). A *p*-value was calculated for each parameter to determine whether change that occurred was statistically significant.

## Results

### Perceptual results

Table 4 depicts the pre-test and post-test scores for each component of the GRBASI scale, as well as the changes noted from pre- to post-test. The perceptual voice quality of 50% of the participants did not change from the pre-test to the post-test. The other 50% of the participants demonstrated a change in perceptual voice quality by no more than one point per parameter.

**TABLE 4:** Perceptual analysis results: Change in scores from pre- to post-test.

Participant	Grade			Roughness			Breathiness			Asthenia/strain			Instability		
	pre	post	change	pre	post	change	pre	post	change	pre	post	change	pre	post	change
2	0	0	0	1	1	0	0	0	0	0	0	0	1	0	1
3	0	0	0	1	1	0	0	0	0	0	0	0	1	0	1
4	0	1	−1	1	1	0	0	0	0	0	0	0	0	1	−1
5	0	0	0	1	1	0	0	0	0	0	0	0	1	0	1
7	0	0	0	0	0	0	0	0	0	0	0	0	0	0	0
9	0	0	0	0	0	0	1	1	0	0	0	0	0	0	0
10	0	0	0	1	1	0	0	0	0	0	0	0	0	0	0
11	0	0	0	0	0	0	0	0	0	0	0	0	0	0	0
12	0	0	0	1	1	0	0	0	0	0	0	0	0	0	0
13	2	0	2	0	1	−1	1	1	0	0	0	0	1	0	1
14	0	0	0	0	0	0	0	0	0	0	0	0	0	0	0
15	0	0	0	0	1	−1	0	0	0	0	0	0	0	0	0

Of the 12 participants in this study, the GRBASI scores (perceptual data) of six (50%) remained the same from the pre-test to the post-test. Of the six participants (the other 50%) who *did* demonstrate changes in GRBASI scores, three received an improved rating of one point (from slight to normal) in instability, but roughness remained the same (slight). One participant was rated normal for hoarseness and instability in the pre-test and was scored as slightly hoarse and unstable in the post-test. One participant received an improved rating of hoarseness and instability but scored as slightly rough in the post-test from a normal score of roughness in the pre-test. Participant 12 was also rated as having slight roughness in the post-test from a normal rating of roughness in the pre-test. No change was noted in the parameters asthenia or strain and breathiness. Two candidates who were rated as slightly breathy in the pre-test were scored the same degree of breathiness in the post-test. One of these participants was part of the 50% who did demonstrate change in GRBASI ratings from pre-test to post-test, whereas the other participant was part of the 50% who demonstrated no change.

### Acoustic results

Table 5 depicts the pre-test and post-test median values for each acoustic parameter measured. The change in median values from pre- to post-test for every parameter is presented. The direction of change is indicated, as is whether or not the direction of change was as expected. A *p*-value is presented, representing whether or not the results are statistically significant.

**TABLE 5:** Acoustic analysis results across all participants.

Measurement	Median	Change	Direction of change	*p*-value
Lowest intensity – pre-test	64	0.5	Expected	0.8062
Lowest intensity –post-test	64.5			
Jitter – pre-test	2.3955	1.0125	Not expected	0.9673
Jitter – post-test	1.383			
Noise/harmonics ratio – pre-test	0.197	0.028	Not expected	0.8867
Noise/harmonics ratio – post-test	0.169			
Maximum phonation time – pre-test	17.415	1.085	Expected	0.3872
Maximum phonation time – post-test	16.33			
s/z ratio – pre-test	1.103	0.03888	Expected	0.0730
s/z ratio – post-test	1.14188			
Highest frequency – pre-test	741.225	22	Expected	0.6182
Highest frequency – post-test	719.225			
Shimmer – pre-test	7.4305	1.094	Expected	0.7256
Shimmer – post-test	8.5245			
Fundamental frequency – pre-test	210.1245	2.9005	Expected	0.1133
Fundamental frequency – post-test	213.025			
Dysphonia Severity Index – pre-test	−0.35	0.25	Expected	0.9807
Dysphonia Severity Index – post-test	−0.6			

For all the measured acoustic parameters a *p*-value indicated that pre-test and post-test parameters did not differ significantly: Lowest intensity, Maximum phonation time, s/z ratio, highest frequency, shimmer, fundamental frequency and the DSI were parameters in which change occurred in the expected direction (the expected direction indicating a decreased effectiveness of voice from pre- to post-test). Noise or harmonics ratio and jitter measurements resulted in a change in the direction that was not expected, indicating results which alluded to improved effectiveness of voice (see Table 2 for a definition of the parameters).

With regard to those parameters that *changed in the expected direction* (decreased effectiveness of voice), lowest intensity demonstrated an increase (i.e. lowest intensity range was reduced) after the experimental condition. Maximum phonation time demonstrated a decrease after the experimental condition. The s/z ratio indicated poorer vocal effectiveness in the post-test. The highest frequency decreased from the pre- to the post-test as expected. An increase in shimmer and fundamental frequency was observed in the post-test measurements. The DSI results indicated that participants were more dysphonic in the post-test than in the pre-test, indicating a diminished voice quality. For the parameters that *changed in the direction not expected*, noise or harmonics ratio demonstrated a decrease in the post-test. Since the values of jitter were in the region of 1%–2% and also decreased, this parameter was not responsible for the shift in the DSI. The implications of the values of jitter indicate that the other variables used to calculate the DSI (MPT, lowest intensity, highest frequency) were responsible for the shift to a more negative DSI value.

## Discussion

### Changes in perceptual and acoustic features of voice

Although not statistically significant, change that was of clinical significance was observed in the acoustic and perceptual parameters of voice in the expected direction.

The change in the s/z ratio and maximum phonation time indicated poorer vocal effectiveness in the post-test in comparison to the pre-test. This reflects a decrease in overall ‘phonatory ability’ and demonstrates a decreased efficiency in subglottic pressure, airflow resistance and vocal fold closure (Wuyts *et al*., 2000, p. 803). The decrease in highest frequency noted in the results often indicates an increased vocal fold mass which hampers vocal fold vibration at higher rates. In addition, the increase in the shimmer value demonstrates the inability of the vocal folds to support a periodic vibration for a distinct period and indicates the existence of turbulent noise in the voice signal (KayPENTAX, 2008). Vocal fatigue and vocal pathology are associated with a decrease in fundamental frequency following a period of voice use (Stemple, Stanley & Lee, 1995; Guimarães & Abberton, 2005; Altman, Atkinson & Lazarus, 2005), as seen in the results of the current study.

The two participants who were rated as mildly breathy in the pre-test were scored as mildly breathy in the post-test. Instability values changed in five participants: of all the parameters demonstrating change, it was the parameter that changed in the highest number of participants from the pre-test to the post-test.

Despite the fact that much of the change in the parameters of voice occurred in the expected direction for both perceptual and acoustic results, there were several unexpected changes in the opposite direction. For perceptual results, an improvement in hoarseness for one participant and in instability for four participants was noted. Acoustic results revealed a decrease in jitter for seven participants, and a decrease in noise or harmonics ratio for seven participants. These changes suggest improved effectiveness of voice, which was not as expected. It is important to consider that the improvements noted may have resulted from a ‘warm-up’ effect. A study conducted by Van Lierde *et al*. (2011) revealed that vocal warm-ups of only 30 minutes significantly increased DSI values. Voice use during the experimental condition may have acted as a form of warm up for voice use and therefore the functioning of the intrinsic and extrinsic laryngeal muscles may have improved. It is likely, then, that improvements in hoarseness and instability, and decreases in jitter and noise or harmonics ratio may have resulted from this warm-up effect. The improvements noted indicate that vocal warm-up exercises may be a tool that should be used by SLPs in conserving their voices.

It should also be taken into consideration that participants’ performance in the post-test may have improved due to their having knowledge of the testing procedures. Participants may have proceeded cautiously in the pre-test but more confidently and comfortably in the post-test.

The results obtained for individual participants were found to coincide with specific participant characteristics that put them at risk for a voice disorder. Poorer vocal effectiveness (as indicated by shimmer, fundamental frequency and highest frequency) was found to be associated with the presence of participant characteristics, including sinusitis, birth control measures, allergies and post-nasal drip.

### Participant characteristics and acoustic change

Fundamental frequency, highest frequency and shimmer showed measurements that suggested decreased vocal effectiveness in a number of participants’ post-test results. In the case of fundamental frequency, seven of the 12 participants demonstrated scores in the post-test that indicated poorer vocal effectiveness. These participants presented with similar characteristics in that four of the seven suffered from sinusitis and were currently implementing birth control. The characteristics associated with poorer vocal effectiveness can be explained as follows: highest frequency was found to be poorer (reduced pitch range) in four of the 12 participants after voice use and this change coincided with the presence of allergies and sinusitis. A combination of sinusitis and post-nasal drip was found to coincide with poorer scores of shimmer for three participants in the post-test. These associations highlight the importance for SLPs to be aware of factors that may contribute to the development of a voice disorder. The treatment and control of these factors will be central to preventative strategies that these professionals may take to preserve their vocal effectiveness.

### Effects of occupational voice use

Overall, the following symptoms were evident in the post-test after all the participants were exposed to the experimental condition: a decrease in vocal effectiveness and efficiency, decreased vocal fold vibration at higher frequencies, impaired ability to maintain periodicity in the amplitude of the voice, vocal fatigue and a diminished voice quality. Although none of the change in any voice parameter was statistically significant, much of it was in the expected direction (decreased vocal effectiveness) and subsequently holds clinical significance. It must be taken into account that over a longer testing period or longer period of voice use, more pronounced results may be obtained. Extended voice use resulting in vocal fatigue or reduced vocal effectiveness may lead to difficulties in controlling voice production and subsequently the voice may deteriorate more quickly or present with more severe dysphonia (Stemple *et al*., 1995). Periods of voice rest have also been found to improve the parameters of voice and have been found to be a valuable addition to voice therapy (Van der Merwe, 2004). The voice use of a professional voice user is not likely to be confined to a two-hour period in a day and typically spans the length of a working day. Considering the change that occurred in vocal effectiveness after only two hours of voice use, it may be inferred that after a typical day of voice use, vocal effectiveness may be diminished to a much larger degree. A period of voice rest lasting 30–60 minutes, combined with appropriate hydration, may restore vocal effectiveness and assist in preventing the development of vocal pathologies (Van der Merwe, 2004; Van Lierde, Claeys, De Bodt & van Cauwenberge, 2007) The implications of the results of this study suggest that the SLP should consider a period of voice rest and hydration after two hours of service delivery. This strategy, consisting of two-hour sessions of service delivery, is consistent with specific guidelines developed for appropriate vocal hygiene and voice conservation such as those set out by Deem and Miller (2000).

Occupationally related voice dysfunction may have a significant impact on job performance, attendance and future career choices (Roy *et al*., 2004). Furthermore, economic burdens may be placed on the professional voice user who displays diminished vocal effectiveness, including costs related to sick leave, voice therapy or surgical management (Roy *et al*., 2004). The implications of reduced effectiveness of voice point to the importance of developing effective prevention and management programmes for SLPs who are either experiencing or at risk of developing reduced effectiveness of voice.

### Limitations and recommendations for future research

This was an exploratory study and further research with this and other populations is necessary. The implications of the results obtained in this study underline the need for future research.

Further research into the effects of voice use during service delivery is necessary because of the importance of voice conservation. This research should be expanded to a larger number of participants in order for results to be more representative of the effects that occur in the real-life everyday use of the SLP. The wide variety of measurements of vocal parameters collected in this study provided a multi-faceted picture of the voice before and after voice use. These measurements are not time consuming to collect and are also collected as a set by the software used. As a set, the measurements provide valuable data. Future research can include the use of all the parameters and measurements used in this study. The experimental condition should be extended to the length of a working day for the effects of voice use to be more representative of typical voice use.

Experimental control should be tightened in future studies. More control over food and drink ingested before the experiment should occur as certain foods may have a negative effect on the parameters of voice (Deem & Miller, 2000). Furthermore, future research should introduce a control group that does not allow hydration during the period of voice use. The amount and type of hydration permitted in the experimental group should be specified and closely monitored. The type of therapy provided by the participants should be more controlled and possibly limited to either adults or children with similar disorders.

## Conclusion

This study has revealed both the positive and the negative effects of voice use during service delivery. The occupational use of voice in SLP students demonstrated a change (whether positive or negative) in the parameters of voice. Although no statistical significance was established, signs which are of clinical significance were observed. These changes may be indicative of the harmful effects of voice overuse, vocal abuse and vocal misuse that are often occupationally required by the SLP.

The importance of vocal hygiene programmes has been brought to light. A suggested strategy is two-hour sessions of service delivery, followed by a period of voice rest and hydration. Vocal hygiene as part of voice conservation may result in prevention, identification and treatment of voice problems before they become significant enough to influence service delivery negatively.
